# Skeletal muscle and metabolic flexibility in response to changing energy demands in wild birds

**DOI:** 10.3389/fphys.2022.961392

**Published:** 2022-07-22

**Authors:** David L. Swanson, Yufeng Zhang, Ana Gabriela Jimenez

**Affiliations:** ^1^ Department of Biology, University of South Dakota, Vermillion, SD, United States; ^2^ College of Health Science, University of Memphis, Memphis, TN, United States; ^3^ Department of Biology, Colgate University, Hamilton, NY, United States

**Keywords:** muscle, hypertrophy, myostatin, IGF-1, myonuclear domain

## Abstract

Phenotypically plastic responses of animals to adjust to environmental variation are pervasive. Reversible plasticity (i.e., phenotypic flexibility), where adult phenotypes can be reversibly altered according to prevailing environmental conditions, allow for better matching of phenotypes to the environment and can generate fitness benefits but may also be associated with costs that trade-off with capacity for flexibility. Here, we review the literature on avian metabolic and muscle plasticity in response to season, temperature, migration and experimental manipulation of flight costs, and employ an integrative approach to explore the phenotypic flexibility of metabolic rates and skeletal muscle in wild birds. Basal (minimum maintenance metabolic rate) and summit (maximum cold-induced metabolic rate) metabolic rates are flexible traits in birds, typically increasing with increasing energy demands. Because skeletal muscles are important for energy use at the organismal level, especially to maximum rates of energy use during exercise or shivering thermogenesis, we consider flexibility of skeletal muscle at the tissue and ultrastructural levels in response to variations in the thermal environment and in workloads due to flight exercise. We also examine two major muscle remodeling regulatory pathways: myostatin and insulin-like growth factor -1 (IGF-1). Changes in myostatin and IGF-1 pathways are sometimes, but not always, regulated in a manner consistent with metabolic rate and muscle mass flexibility in response to changing energy demands in wild birds, but few studies have examined such variation so additional study is needed to fully understand roles for these pathways in regulating metabolic flexibility in birds. Muscle ultrastrutural variation in terms of muscle fiber diameter and associated myonuclear domain (MND) in birds is plastic and highly responsive to thermal variation and increases in workload, however, only a few studies have examined ultrastructural flexibility in avian muscle. Additionally, the relationship between myostatin, IGF-1, and satellite cell (SC) proliferation as it relates to avian muscle flexibility has not been addressed in birds and represents a promising avenue for future study.

## Introduction

Phenotypically plastic responses of morphology, behavior and physiology to environmental variation are ubiquitous among living organisms (Pigliucci 2005; Morel-Journel et al., 2020; Sommer 2020). Genetic variation for plastic responses to environmental variation occurs among organisms (Pigliucci 2005), such that individual organisms differ predictably in plastic responses, including plasticity of metabolic rates (Norin and Metcalfe 2019). Phenotypic plasticity includes developmental plasticity, where environmental conditions experienced during development impact adult phenotypes, and reversible plasticity (i.e., phenotypic flexibility, Piersma and Drent 2003), where adult phenotypes can be reversibly altered according to prevailing environmental conditions. Such flexible responses allow better matching of adult phenotypes to the environment and can generate positive fitness benefits (Piersma and Van Gils 2011; Petit et al., 2017; Latimer et al., 2018).

The primary functions of skeletal muscle in birds are locomotion and thermogenesis. Powered flight is supported by large flight muscles, which comprise 10%–25% of total body mass in volant birds (Hartman 1961; DuBay et al., 2020). For more terrestrial species, leg muscles comprise larger fractions of total musculature (Hartman 1961; Bennett 1996). Thermogenesis in birds may be accomplished by both shivering and non-shivering mechanisms, and, although evidence continues to accumulate for muscular non-shivering thermogenesis in chicks or growing birds (for reviews see Rowland et al., 2015), thermogenesis in adult birds appears to occur primarily through shivering (Hohtola 1982; Cheviron and Swanson 2017; Stager and Cheviron 2020). Changing energetic demands for locomotion or thermoregulation throughout the annual cycle of birds, such as migration or cold winters, may result in flexible changes to the volume, ultrastructure, or aerobic capacity of skeletal muscles (Dawson et al., 1983; Marsh and Dawson 1989; Swanson 2010; Jimenez 2020).

### Organismal metabolic and flight muscle flexibility in response to temperature

Birds resident in highly seasonal climates typically upregulate their maximum capacity for thermogenesis, known as summit metabolic rate (M_sum_), in winter (Swanson 2010; Swanson and Vézina 2015). This elevation in M_sum_ is associated with improved capacities for cold tolerance and endurance under submaximal levels of cold exposure (Marsh and Dawson 1989; Swanson 2001; Swanson and Liknes 2006). In addition, M_sum_ is positively related to overwinter survival for small birds in cold climates (Petit et al., 2017; Latimer et al., 2018). Basal metabolic rate (BMR, miniumum maintenance metabolic rate) also often increases in winter in birds from cold climates or with cold acclimation in birds, although this trend is far from universal (Marsh and Dawson 1989; Swanson 2010, Table 1). As a recent example, Li et al. (2020) documented a 102% increase in BMR in winter relative to late spring in Eurasian tree sparrows (*Passer montanus*) from the relatively mild winter climate of southeastern China. BMR may also be associated with overwinter survival in birds, but on a fluctuating basis, with high BMR favored in cold winters and low BMR favored in warm winters (Nilsson and Nilsson 2016).

**TABLE 1:** Results of recent studies (since 2010) of natural seasonal or migratory acclimatization, temperature acclimation, or experimental flight training effects on pectoralis/flight muscle mass or size and metabolic rates (BMR or M_sum_) in birds.

Species	Acclimatization or acclimation condition	Trend in pectoralis muscle mass or size	Trend in metabolic Rates	Reference
Black-capped chickadee	Seasonal acclimatization to cold winter	Dry mass increase by 10%	M_sum_ increase by 22%	Petit et al. (2014)
*Poecile atricapillus*				
Snow bunting	Outdoor captive all year in winter range	Ultrasound thickness increase by 8%	M_sum_ increase by 23%	Le Pogam et al. (2020)
*Plectrophenax nivalis*				
Chinese bulbul	Seasonal acclimatization to cool winter	Dry mass increase by 12%	BMR increase by 21%	Zheng et al. (2014)
*Pycnonotus sinensis*				
Chinese hwamei	Acclimated to 15 and 35°C	Dry mass increase by 20%	BMR increase by 40%	Zhou et al. (2016)
*Garrulax canorus*				
Eurasian tree sparrow	Acclimated to 10 and 30°C; varying photoperiod	No sig. change in dry mass of skeletal muscle	BMR increase by 45%	Li et al. (2020)
*Passer montanus*				
Chinese bulbul	Seasonal acclimatization to cool winter	No sig. change in dry mass	BMR increase by 42%	Wang et al. (2019)
*Pycnonotus sinensis*				
Chinese hwamei	Acclimated to 10 and 30°C; varying photoperiod	No sig. change in dry mass	BMR increase by 18%	Hu et al. (2017)
*Garrulax canorus*				
Dark-eyed junco	Acclimated to 3 and 24°C; varying photoperiod	No significant change in wet mass	M_sum_ increase by 18%	Swanson et al. (2014a)
*Junco hyemalis*				
White-throated sparrow	Acclimated to -8 and 28°C	No sig. change in dry mass	M_sum_ increase by 19%	Barcelo et al. (2017)
*Zonotrichia albicollis*			BMR increase by 15%	
Black-capped chickadee	Acclimated to -10 and 27°C	No sig. change in dry mass	M_sum_ increase by 20%	Milbergue et al. (2018)
*Poecile atricapillus*			BMR increase by 5%	
Snow bunting	Seasonal acclimatization cold winter, Arctic summer	Winter ultrasound thickness increase by 3.1%	No significant change in M_sum_	Le Pogam et al. (2021)
*Plectrophenax nivalis*				
White-browed sparrow-weaver	Seasonal acclimatization to mild winter	Dry mass increase by 5%	M_sum_ decrease by 26%	Noakes et al. (2020)
*Plocepasser mahali*				
House sparrow	Acute, repeated exposure to severe cold	Wet mass increase by 5%	M_sum_ increase by 14.6%	Zhang et al. (2015)
*Passer domesticus*			BMR increase by 10.3%	
Gray catbird	Natural migratory acclimatization	Wet mass increase by 8.7% in migrants	M_sum_ increase by 19-29%	DeMoranville et al. (2019)
*Dumetella carolinensis*				
Warbling vireo	Natural migratory acclimatization	Wet mass increase by 10.3% in spring	M_sum_ increase by 18.3%	King et al. (2015)
*Vireo gilvus*				Swanson (1995)
Yellow warbler	Natural migratory acclimatization	Wet mass increase by 14.7% in spring	M_sum_ increase by 23.3%	King et al. (2015)
*Setophaga petechia*				Swanson and Dean (1999)
Yellow-rumped warbler	Natural migratory acclimatization	No significant seasonal change in wet mass	M_sum_ increase by 19.8% in spring vs. fall	King et al. (2015)
*Setophaga coronata*				Swanson and Dean (1999)
Black-capped chickadee	Feather clipping to increase flight costs	Muscle score increase	M_sum_ increase by 17%	Petit and Vézina (2014a)
*Poecile atricapillus*				
House sparrow	Experimental flight training	Wet mass increase by 7%	M_sum_ increase by 15.5%	Zhang et al. (2015)
*Passer domesticus*			BMR decrease by 37.9%	

For birds wintering in warmer subtropical or tropical climates, seasonal patterns of variation in M_sum_ are much more variable (McKechnie et al., 2015), with winter increases, winter decreases or no seasonal change evident for various bird species (Smit and McKechnie 2010; Wells and Schaeffer 2012; Van de Ven et al., 2013; Thompson et al., 2015; Noakes et al., 2017; Pollock et al., 2019; Noakes and McKechnie, 2020). This suggests that thermoregulatory demand is an important determinant of M_sum_ in birds (Swanson and Garland 2009; Stager et al., 2016; Stager et al., 2020; Stager et al., 2021). The migratory condition (especially the more rapid spring migration; Nilsson et al., 2013; Horton et al., 2016; Schmaljohann et al., 2017) in birds also appears to generally result in an upregulation of M_sum_, perhaps as a by-product of enhancement of flight capacities (Swanson 1995; Swanson and Dean 1999; Vézina et al., 2007; Petit and Vézina 2014a; Corder and Schaeffer 2015).

Because M_sum_ is primarily a function of skeletal muscle metabolism, increases in M_sum_ are likely mediated by changes in skeletal muscle. Increases in M_sum_ at the organismal level can be accomplished by changes in either the size of the skeletal muscles involved in thermogenesis or in the cellular metabolic intensity of individual muscle fibers (Swanson 2010). For birds, the prevailing dogma is that changes in muscle mass (especially the pectoralis, which is the largest muscle in the body of volant birds and, therefore, the principal thermogenic organ, Marsh and Dawson 1989) are regular contributors to changes in M_sum_, but that variation in cellular metabolic intensity is a less consistent mediator of flexibility of M_sum_ in response to changing energy demands associated with seasonal thermogenesis or migration (Swanson and Vézina 2015).

**FIGURE 1:**
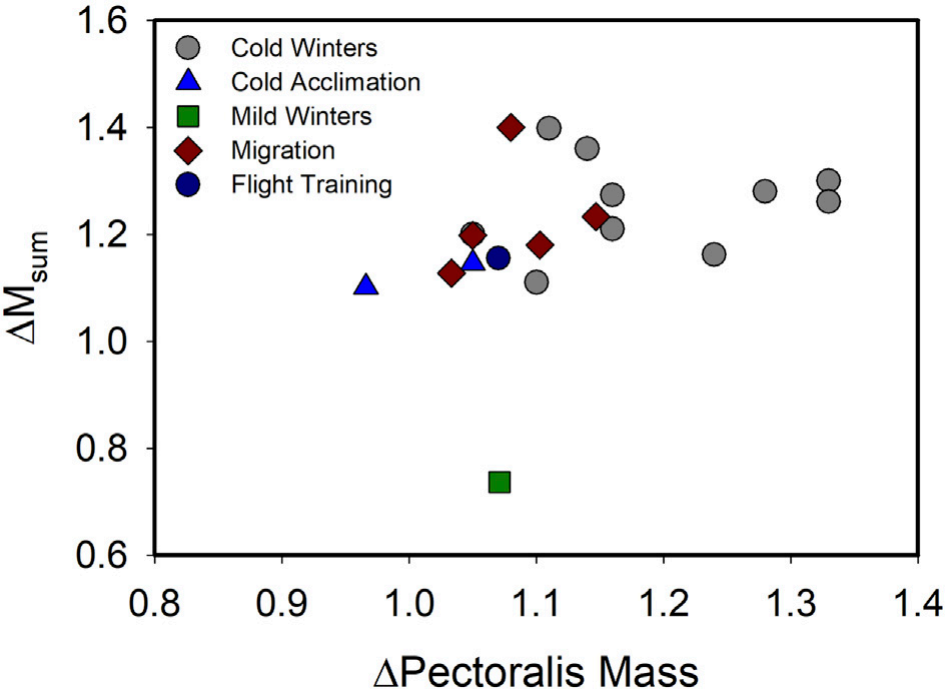
Relationship of variation in pectoralis muscle mass (ΔPectoralis Mass) with variation in summit metabolic rate (ΔM_sum_) across a range of natural acclimatization (winter *vs.* summer in climate with cold or mild winters, migratory *vs.* non-migratory conditions) and experimental acclimation (cold *vs.* warm, flight training *vs.* control) for birds. A linear regression on these data indicated no significant relationship (*F*_1,17_ = 1.918, *p* = 0.184), including after removal of the “Mild Winters” data point (*F*_1,16_ = 2.775, *p* = 0.115), suggesting that despite increases in both M_sum_ and pectoralis muscle mass often occurring under conditions of increasing energy demand in birds, the two traits are not tightly coupled. Data from Swanson (1990), Swanson (1991), Swanson (1995), O’Connor (1995a), O’Connor (1995b), Cooper (2002), Cooper and Swanson (1994), Petit et al. (2014), Liknes and Swanson (2011), Sgueo et al. (2012), Swanson et al. (2014a,b), Milbergue et al. (2018), Noakes et al. (2020), DeMoranville et al. (2019), King et al. (2015), Zhang et al. (2015), Vézina et al. (2006).

Recent evidence, however, suggests that, although muscle hypertrophy is still a common correlate of increases in BMR and M_sum_ at the organismal level, that tight coupling between muscle hypertrophy and increases in M_sum_ is not as consistent as previously believed (Figure 1; Table 1). BMR is positively correlated with pectoralis mass or flight muscle mass for many bird species, including house sparrows (Chappell et al., 1999) and non-breeding European starlings (Vézina and Williams 2003). Several recent studies also document the expected positive relationships between pectoralis muscle mass and BMR or M_sum_. For example, pectoralis muscle mass was positively correlated with M_sum_ in summer-acclimatized captive American goldfinches (*Spinus tristis,* Swanson et al., 2013). In addition, even when acclimation- or acclimatization-induced changes in pectoralis or skeletal muscle masses are not concordant with similar changes in M_sum_ or BMR (Table 1), muscle masses and metabolic rates may still show positive correlations (Barcelo et al., 2017; Milbergue et al., 2018; Li et al., 2020). These data suggest that muscle masses, especially mass of the flight muscles, are important determinants of organismal metabolic rates. In addition to changes in muscle masses, changes in muscle ultrastructure in response to winter or cold may also be evident. For example, black-capped chickadees acclimated to −5°C had 23% larger pectoralis fiber diameter than birds acclimated to 20°C (Vézina et al., 2020). When birds in these temperature treatments were exposed to a 3 h temperature drop of 15°C, cold-acclimated birds increased the number of capillaries per muscle fiber and the number of nuclei per fiber by 22%, whereas no changes occurred in warm-acclimated birds, suggesting that cold-acclimated birds had an increased capacity for response to an acute temperature decrease (Vézina et al., 2020), but how these changes relate to acute changes in M_sum_ is not known.

In contrast to these studies, a number of other recent studies suggest that seasonal or cold-induced changes in muscle size do not necessarily accompany changes in metabolic capacities in birds (Table 1). In addition to study results summarized in Table 1 showing a lack of congruence in seasonal or cold-induced variation in muscle masses and organismal metabolic rates, Bai et al. (2016) found no significant correlations between pectoralis mass residuals and BMR residuals in brambling (*Fringilla montifringilla*), little bunting (*Emberiza pusilla*), and Eurasian tree sparrow (*P. montanus*). The overall generalization from recent work on flexibility in pectoralis mass and metabolic capacities in birds in response to winter or cold exposure is well-stated by Milbergue et al. (2018), who noted in the title of their paper that “large muscles are beneficial but not required for improving thermogenic capacity in small birds.” Such a result indicates that cellular and biochemical adjustments, such as alterations of the number of mitochondria, aerobic enzyme activities, substrate transport pathways, and, potentially, mechanisms promoting muscular non-shivering thermogenesis, can also contribute to changes in organismal thermogenic capacity in response to temperature (Swanson 2010; Milbergue et al., 2018).

### Organismal metabolic and flight muscle flexibility in response to migration or flight training

In addition to winter acclimatization or cold acclimation influences on muscle masses and metabolic rates in birds, development of migratory disposition is also typically associated with increases in flight muscle mass of up to 35% (Swanson 2010; Piersma and Van Gils 2011). Such increases in flight muscle mass can occur over periods as short as a few days and apparently do not require exercise as a cue for hypertrophy (Dietz et al., 1999; Piersma and Van Gils 2011). Examples of such rapid hypertrophy associated with migration include an approximately 19% increase in lean dry pectoralis muscle mass over the last few days of a 24-days stopover in red knots (*Calidris canutus,* Piersma et al., 1999). Similarly, Bar-tailed godwits (*Limosa lapponica*) showed increases in lean dry pectoralis mass of 21 and 27% in males and females, respectively, between early and late stopover phases (Landys-Ciannelli et al., 2003). More recent studies also generally support the trend of muscle hypertrophy during the migratory period for birds. For example, pectoralis muscle mass and fiber diameter both increased (by approximately 17 and 35%, respectively) with development of migratory condition in outdoor captive Snow buntings (Vézina et al., 2021). Concurrently with this change, myonuclear domain (MND) also increased during migration (Vézina et al., 2021; more on this below). Velten et al. (2016) compared winter flocks of white-crowned sparrows (*Zonotrichia leucophrys*) in central California with birds just arriving from migration in May in Alaska and found that flight muscle mass was greater upon arrival in Alaska than during winter or just prior to departure in April in California. Flight training also typically results in increases in pectoralis muscle mass in birds. Fight-trained European starlings (*Sturnus vulgaris*) increased pectoralis mass by 8% compared to untrained birds (DeMoranville et al., 2020). Zhang et al. (2018a) forced Zebra finches (*Taeniopygia guttata*) to hover while feeding, thus increasing flight costs, which resulted in a 9% increase in pectoralis mass for females, but not for males.

Oftentimes, these increases in pectoralis mass with migration or flight training are correlated with increases in metabolic capacities in birds (Table 1). For example, M_sum_ was positively correlated with breast muscle size measured by ultrasonography in migratory and cold acclimated red knots (Vézina et al., 2006; Vézina et al., 2007). In addition, male Ruby-crowned kinglets (*Regulus calendula*) elevated M_sum_ by 10.9% between spring and fall migration, although female kinglets showed statistically stable M_sum_ between spring and fall, consistent with a more rapid pace of migration in spring (Swanson and Dean 1999). Results from recent studies are also generally consistent with elevated pectoralis mass being positively associated with elevations in M_sum_, although this does not occur for all birds studied (Table 1).

## The signaling pathways regulating muscle mass

**FIGURE 2:**
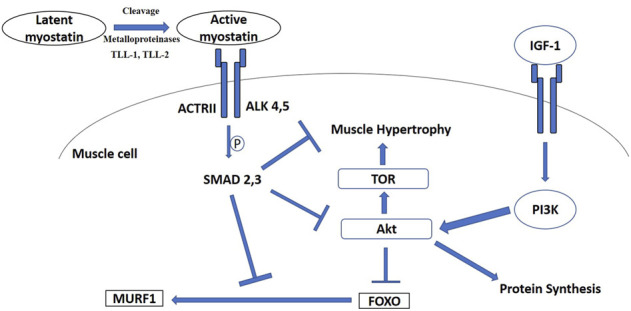
Signaling diagram illustrating myostatin and insulin-like growth factor—1 (IGF-1) pathways. ActRII, activin receptor II; ALK, type I activin receptors; Akt, protein kinase B; PI3K, phosphatidylinositol 3-kinase; TOR, target of rapamycin; TLL, Tolloid-like protein; FOXO, Forkhead Box-O; MURF1, muscle-specific E3 ubiquitin ligase muscle RING-finger 1.

As noted above, one major factor often supporting increases in thermogenic and exercise capacity in small birds is pectoralis muscle hypertrophy (O'Connor 1995a; Swanson, 2010; Swanson and Merkord, 2013; Swanson et al., 2013). However, the signaling pathways and cellular and molecular mechanisms regulating muscle and organ mass changes underlying phenotypic flexibility in wild, free-living, birds have received relatively little research attention to date (Swanson, 2010). Two major candidates for signaling pathways regulating flexibility in muscle mass are myostatin and insulin-like growth factor -1 (IGF-1) pathways (Favier et al., 2008; Hennebry et al., 2017). While myostatin acts by inhibiting muscle growth, IGF-1 signals an increase in protein synthesis and, therefore, an increase muscle mass (Figure 2; Hennebry et al., 2017). The interaction of these signaling pathways with cellular and molecular mechanisms influencing muscle growth and function are important determinants of muscle mass and should be tightly regulated in birds (Lozier et al., 2018; Sartori et al., 2021).

### Myostatin signaling pathway

Of these two major pathways, the myostatin signaling pathway is better studied in avian phenotypic flexibility. Myostatin (MSTN), also known as growth differentiation factor 8 (GDF-8), is a TGF-β superfamily growth factor (McPherron et al., 1997; Lee, 2004; Sharma et al., 2015). Myostatin is a potent autocrine/paracrine inhibitor of muscle growth in mammals and birds, and is a highly conserved protein, with the active peptide being identical in birds and mammals, such as mice, rats, humans, pigs, dogs, chickens and turkeys (Lee, 2004; McFarland et al., 2006; McFarland et al., 2007). Similar to other TGF-β superfamily members, myostatin is synthesized in skeletal muscle in an inactive form (52 kDa) that requires two proteolytic cleavages for activation (McPherron et al., 1997; Lee and McPherron, 2001). An initial cleavage of the propeptide from mature myostatin by furin or other propeptide convertases produces a latent form with the propeptide non-covalently bound to the mature myostatin (Lee, 2008; Pirruccello-Straub et al., 2018). Cleavage of the NH_2_-terminal propeptide domain from mature myostatin results in formation of the active COOH-terminal dimer, which binds to myostatin receptors and is required for myostatin activity (Thomas et al., 2000; Lee, 2008). Metalloproteinases, including BMP-1/tolloid family members TLL-1 and TLL-2, can perform this second cleavage to activate myostatin (Wolfman et al., 2003). Because of these enzymatic cleavages, a 36/40 kDa Latency Associated Peptide and a 12.5/26 kDa mature peptide, corresponding to a C-terminal monomer or dimer respectively, have been observed (Thomas et al., 2000; Lee and McPherron, 2001; McFarlane et al., 2005). Both mature and LAP forms of myostatin are secreted into the circulation (Lee and McPherron, 2001; Hill et al., 2002), allowing myostatin to function in an autocrine, paracrine, or endocrine manner.

Active mature myostatin binds to the type II activin receptors A and B (ActRIIA and ActRIIB) (Lee et al., 2005), which recruit and activate type I activin receptors four and five (Alk4 and Alk5) (Rebbapragada et al., 2003). These events stimulate phosphorylation and activation of the transcriptional factors Smad2 and Smad3, which join with Smad4 into a Smad2/3/4 complex that triggers gene transcription (Figure 2; Zhu et al., 2004). Additionally, myostatin decreases Akt (protein kinase B) phosphorylation (Trendelenburg et al., 2009), which is accompanied by the accumulation of dephosphorylated active Forkhead Box-O1 (FOXO1) and FOXO3 (Allen and Unterman, 2007), followed by upregulation of components of the ubiquitin-proteasome pathway, such as atrogin-1 and the muscle-specific E3 ubiquitin ligase muscle RING-finger1 (MURF1) (McFarlane et al., 2006) (Figure 2). As one of the master regulators of muscle mass and protein synthesis, regulation of myostatin could also occur at the post-transcriptional (regulating microRNAs) (Clop et al., 2006) and post-translational (regulating protein-protein interaction) levels (Sharma et al., 2015). Moreover, myostatin could also promote quiescence of satellite cells in skeletal muscle so that they fail to become incorporated into the muscle fiber and inhibit self-renewal of satellite cells (McCroskery et al., 2003; McFarland et al., 2007).

Mutations in the myostatin gene and blockage of myostatin action result in dramatic increases in muscle growth (McPherron and Lee, 1997; Lee, 2004; Chen and Lee, 2016). In mammals, treatment of adult mice with an inhibitory antibody to myostatin resulted in muscle hypertrophy, suggesting that myostatin is an important mechanism for muscle remodeling in adult animals (Whittemore et al., 2003). In poultry, myostatin tightly regulates pectoralis muscle mass and inhibition of myostatin dramatically increased pectoralis mass in chicken, turkey, domestic pigeon, duck, and Japanese quail (McFarland et al., 2006; Xu et al., 2013; Shin et al., 2015; Liu et al., 2019; Zhao et al., 2019; Kim et al., 2020). Phenotypic flexibility in wild, free-living, birds could be more complicated since birds can modify flight muscle independent of workload (Swaddle and Biewener, 2000) and changes in flight muscle mass can occur without training (Dietz et al., 1999). Because of the prominent role of myostatin in muscle remodeling, it is reasonable to hypothesize that myostatin is involved in phenotypic flexibility of muscle mass in birds generally, potentially playing roles in muscle size changes associated with different periods of the annual cycle (e.g., winter acclimation, migration, molt) where phenotypic flexibility of muscle mass occurs.

### Myostatin in avian phenotypic flexibility

Investigation of a role for myostatin in avian metabolic flexibility was first examined in house sparrows during winter acclimatization where increases in pectoralis muscle mass occurred in response to winter cold (Swanson et al., 2009). In this study, myostatin and TLL-1 gene expression were both lower in pectoralis muscle of house sparrows during winter than during summer, suggesting reductions in myostatin levels and myostatin processing capacity in winter consistent with winter increases in muscle mass and metabolic capacities. However, in a follow-up study (Swanson et al., 2014b), results for both American goldfinches (*S. tristis*) or black-capped chickadees (*Poecile atricapillus*) between summer and winter were not consistent with house sparrows. Swanson et al. (2014b) found that pectoralis muscle mass increased in winter for naturally acclimatized American goldfinches (by 15%) but not for black-capped chickadees, despite chickadees typically showing winter increases in pectoralis mass and M_sum_ (Cooper and Swanson, 1994; Liknes and Swanson, 2011; Petit and Vézina, 2014b; Petit et al., 2014). Myostatin gene or protein expression did not vary significantly between summer and winter for goldfinches, although there was a tendency toward lower levels in winter, but both TLL-1 and TLL-2 were reduced in winter goldfinches, suggesting a lower capacity for processing and activation of myostatin in winter consistent with a winter increase in pectoralis muscle mass (Swanson et al., 2014b). For chickadees, myostatin gene and protein expression showed only non-siginificant decreases and TLL-1 also showed no significant seasonal variation, but TLL-2 exhibited a significant winter decrease (Swanson et al., 2014b). Thus, although changes in muscle mass and expression of the myostatin system were generally consistent with a role for the myostatin pathway in regulating seasonal changes in muscle mass and thermogenic capacity in chickadees and goldfinches, these changes were not uniform for both species.

Swanson et al. (2017) examined within-winter variation in pectoralis muscle mass and expression of the myostatin system in dark-eyed juncos and house sparrows and found that pectoral muscle mass residuals were positively correlated with Short-term (ST; 0–7 days prior to measurement) temperature variables for both species, which was opposite of predictions and suggests that cold temperatures resulted in catabolism of skeletal muscles over the short term. Pectoralis gene or protein expression of myostatin and the TLL proteases were only weakly correlated with ST and medium-term (MT; 14–30 days prior to measurement) temperature variables, and myostatin expression was negatively related with ST and MT temperatures for juncos but positively related with long-term (30-years average temperatures) and MT temperatures for house sparrows (Swanson et al., 2017), so no consistent regulation of the myostatin system in response to acute temperature variation within winters was evident, despite within-winter variation in basal and summit metabolic rates in response to ST and MT temperature variables in small birds (Swanson and Olmstead, 1999).

For migration-induced muscle mass changes, myostatin gene expression did not vary significantly during the migratory season (spring and fall) compared to the non-migratory season (winter) in white-throated sparrows (*Zonotrichia albicollis*) (Price et al., 2011). Moreover, pectoralis and heart mRNA expression of myostatin, TLL-1 and TLL-2 also did not differ significantly among seasons for yellow warblers (*Setophaga petechia*), warbling vireos (*Vireo gilvus*), and yellow-rumped warblers (*Setophaga coronata*), either between migratory seasons (spring of fall) or compared to summer (King et al., 2015). In contrast, myostatin protein levels in pectoralis muscle were lowest during the spring migratory season, concomitant with the greatest pectoralis muscle mass for all three species (King et al., 2015). Similar to winter acclimatization, these data offered mixed support for a regulatory role for myostatin in supporting increases in muscle masses during migration and suggest that patterns of gene and protein expression of the myostatin system do not vary in lockstep. These results further suggest a potentially important role for post-transcriptional regulation of the myostatin pathway in bird responses to seasonally changing energy demands.

Other than natural winter and migratory adjustments, experimental photoperiod, temperature and flight training treatments have been employed to examine a potential role for myostatin in avian metabolic flexibility. Photoperiod (short- *vs.* long-day) and temperature (3°C or 24°C) treatments did not significantly alter gene expression of myostatin or the TLLs nor myostatin protein levels in dark-eyed juncos (*Junco hyemalis*) (Zhang et al., 2018a). Photoperiod-stimulated white-throated sparrows showed higher myostatin mRNA expression for long-day (migratory) compared to short-day (winter) treatments, despite long-day sparrows having greater flight muscle dry mass (Price et al., 2011). In addition to regulating muscle mass, myostatin is also known to increase fat accumulation in adipose tissue in mammals (Lin et al., 2002; McPherron and Lee, 2002; Rebbapragada et al., 2003) by either directly acting on receptors on adipocytes or indirectly saving energy from decreased musculature (Guo et al., 2009). Consequently, elevated myostatin levels might function in adipogenesis in photo-stimulated birds in migratory disposition, which must gain fat mass in preparation for migration.

Compared with seasonal changes, myostatin’s role in muscle remodeling in birds during experimental manipulation was more consistent. Experimental increases in foraging costs of zebra finches (*T. guttata*), accomplished by forcing birds to forage while hovering, resulted in female, but not male, finches showing lower myostatin protein levels than controls (Zhang et al., 2018b). It is important to note here that female zebra finches average shorter wings and higher wing loading than males, so increased flight costs might be expected to disproportionately affect females. Indeed, only female zebra finches in the high foraging group in this study showed reduced total fat masses and increased pectoralis muscle masses, whereas male finches had similar fat and pectoralis mass between groups (Zhang et al., 2018b). Gene expression of myostatin and the TLLs, however, did not vary significantly after high foraging cost treatments for either sex. Other than experimentally increasing foraging costs, flight exercise training has been employed to study the role of myostatin in avian muscle remodeling. Two-week incremental wind tunnel flight training did not significantly modify myostatin or TLL-1 gene expression in European starlings (*S. vulgaris*) (Price et al., 2011). However, this training protocol also did not significantly affect flight muscle mass after controlling for body mass. In another study, Zhang et al. (2015) used experimental cold exposure and exercise training protocols in house sparrows and both training protocols increased pectoralis muscle mass and reduced pectoralis myostatin protein levels. However, gene expression of pectoralis myostatin did not change significantly and gene expression of the TLLs increased for both training protocols. Again, these studies offer partial support for the hypothesis that the myostatin system plays a regulatory role in muscle mass changes in response to changing energy demands in birds and suggest that gene and protein expression of the myostatin system do not necessarily vary in tandem, highlighting again the potential importance of post-transcriptional regulation of this system.

### IGF-1 signaling pathway

In addition to myostatin, the growth hormone/Insulin-like growth factor 1 (IGF-1) axis stimulates growth and accounts for up to 83% of postnatal growth and skeletal muscle hypertrophy in young mammals (Hennebry et al., 2017). Locally produced IGF-1 is now considered to have the predominant influence on tissue growth and local delivery of IGF-1 to skeletal muscle is essential for muscle hypertrophy (Shavlakadze et al., 2010). IGF-1 activates the IGF-1 receptor and phosphorylates PI3 kinase (PI3K) and Akt (Figure 2; Rommel et al., 2001). This process stimulates protein synthesis in myocytes and also satellite cell proliferation, leading to hypertrophy (Snijders et al., 2015). As mentioned above, the PI3K/Akt pathway is also common to the myostatin system, so there appears to be cross-regulation between myostatin and IGF-1 (Figure 2; Morissette et al., 2009; Yoshida and Delafontaine, 2020). However, the myostatin knockout mouse model also suggested that IGF-1 could stimulate muscle hypertrophy in the absence of myostatin, indicating distinct mechanisms for myostatin and IGF-1 regulation of skeletal muscle mass (Hennebry et al., 2017). IGF-1 also promotes skeletal muscle growth through the PI3K/Akt pathway in chickens (Deng et al., 2014; Yu et al., 2015; Nakashima and Ishida, 2018; Nakashima and Ishida, 2019). Even though the IGF-1 axis is a prime target for muscle remodeling in poultry (e.g., Tomas et al., 1998; Saneyasu et al., 2016; Saneyasu et al., 2017; Chen et al., 2018), the IGF-1 axis has received much less research attention in wild birds, with reference to muscle remodeling for phenotypic flexibility, compared with the myostatin signaling pathway. Adélie penguins chicks (*Pygoscelis adeliae*) expressed high IGF-1 mRNA in pectoralis muscle during a rapid growth period, indicating that it plays an important role in the development of an enhanced muscle phenotype (Dégletagne et al., 2013). Gene expression of IGF-1 increased in the pectoralis muscle of migratory Gambel’s white-crowned sparrows (*Zonotrichia leucophrys gambelii*), but decreased in the gastrocnemius muscle at pre-departure stage (Pradhan et al., 2019). On the other hand, IGF-1 mRNA expression in pectoralis of white-throated sparrows was stable during migratory seasons (spring and fall) compared to winter (Price et al., 2011). Flight-trained European starlings showed higher IGF-1 mRNA expression than untrained controls, even though flight muscle mass did not vary significantly between these two groups (Price et al., 2011). Taken together, these data suggest that the IGF-1 signaling pathway might play an important role, independent of myostatin, in modulating avian phenotypic flexibility, but the results to date remain tentative and inconclusive due to limited data.

In addition, transcriptomic analyses on dark-eyed juncos under different temperature and photoperiod treatments indicated that very few genes associated with the mammalian target of rapamycin (mTOR) signaling pathway that governs protein synthesis, and with which myostatin and IGF-1 interact (Amirouche et al., 2009; Ma et al., 2018), were changed, and those genes were counterintuitively upregulated in warm-acclimated birds (Stager et al., 2015). Cheviron and Swanson (2017), however, examined seasonal transcriptomic variation in naturally seasonally acclimatized black-capped chickadees and American goldfinches and found winter upregulation in *mTOR* and genes in the BMP-signaling cascade (*TOB1* and *BMPR2*) in pectoralis muscle, suggesting possible involvement of these pathways in seasonal phenotypic flexibility.

## Ultrastructural changes associated with flexibility in muscle mass

### Myonuclear domain and its significance to muscle function

Skeletal muscle is a multinucleated syncitium. As such, nuclei are each supported by linker of nucleoskeleton and cytoskeleton (LINC) complexes that preserve nuclear positioning (Azevedo and Baylies 2020), so as to minimize transport distances (Snijders et al., 2020). This multinucleation develops through a well-coordinated and seemingly conserved process in vertebrates (Azevedo and Baylies 2020). Progenitor myoblasts are first specified in the embryonic mesoderm to undergo cell-cell fusion events that increase nuclear and cytoplasmic mass to form syncytial myotubes (Azevedo and Baylies 2020). Through myogenesis, these nascent myotubes undergo changes and movements to develop into mature myofibers with specifically determined nuclear positioning and internuclear distances (Azevedo and Baylies 2020). However, not all myoblasts participate in embryonic myogenesis. Such “unfused” myoblasts are termed satellite cells (SCs) and constitute a multipotential mesenchymal stem cell population with the ability to undergo myogenesis or alternative trans-differentiation programs (Velleman 2015). Though undifferentiated, SCs are heterogenous in that distinct sub-populations express a particular set of cell surface markers, differ in activation kinetics, and exhibit unique self-renewal timeframes that make them fiber-type specific and dictate their ability to proliferate and differentiate during post-embryonic muscle growth and regeneration (Velleman 2015; Forcina et al., 2019; Jankowski et al., 2020). In birds, as well as other vertebrates, a SC population surrounds mature myofibers and is key to enabling the post-hatch growth and regeneration of terminally differentiated myofibers in response to mechanical stimuli, injury, and homeostatic factors, including environmental changes (Mauro 1961; Kuang et al., 2007; Forcina et al., 2019). Protected between the basal lamina and sarcolemma of each muscle fiber that preserves their survival and niche behavior, SCs remain in a non-growing, quiescent state with low transcriptional activity until they receive the appropriate signals that restore their proliferative activity by stimulating their re-entrance into the cell cycle (Mauro 1961; Forcina et al., 2019). Then, they can undergo symmetric and asymmetric divisions that maintain the stem cell pool while simultaneously producing cells with reactivated transcriptional expression that allow for differentiation and contribution to muscle plasticity (Kuang et al., 2007; Forcina et al., 2019). Afterwards, they can fuse with existing myofibers to add nuclei, enhance diameter and length, and augment protein synthesis potential through hypertrophic accretion (Forcina et al., 2019; Jankowski et al., 2020; Murach et al., 2021), otherwise DNA may become limiting in larger cells (Cramer et al., 2020). Most muscle growth in adult organisms happens via hypertrophy, that is, an increase in muscle fiber diameter (Kinsey et al., 2007). Because muscle is a post-mitotic, multinucleated tissue, new adult muscle growth via hypertrophy may necessitate either an upregulation of the protein synthesis machinery by existing myonuclei or new nuclei to be drawn into the muscle fiber from a population of SCs. Each myonucleus in a muscle fiber is responsible for servicing a certain volume of cytoplasm known as a myonuclear domain (MND) (Rosser et al., 2003; Qaisar and Larsson, 2014). The cytoplasm of a muscle fiber must be highly organized to compartmentalize the necessary metabolic and contractile machinery. One could, therefore, think of the regulation of muscle fiber diameter and/or cross-sectional area in terms of the balance between production and degradation of cytoplasmic components (Hughes and Schiaffino, 1999; Van der Meer et al., 2011).

Other circulating factors that may activate proliferation of SC in non-injury states into myofibers include growth hormone, follistatin (a myostatin antagonist), and IGF-1, among others (Forcina et al., 2019; Murach et al., 2021). Additionally, increases in lactate concentrations may act as a signaling molecule for increased proliferation of SCs (Nalbandian et al., 2020), and others have suggested that glycolytic enzymes are necessary for optimal muscle growth, at least in *Drosophila* models (Graca et al., 2021). However, it is unclear how regulation to either add new nuclei or upregulate existing myonuclear activity is accomplished within muscle fibers (Cramer et al., 2020).

### Myonuclear domain and muscle remodeling in mammals with respect to energy demand

MND regulation is much more extensively studied in mammals than in birds, so an exploration of mammalian regulation of MND can provide the necessary background to examine similar MND regulation in birds. In mammals, hypertrophy can be initiated and sustained for a time in the absence of SCs in adult mice, but hypertrophy maintained for 8 weeks or more is dampened without additional SCs (Murach et al., 2021), and large changes to MND during muscle hypertrophy do not persist (Snijders et al., 2020). In mammals, myonuclear number is positively correlated with muscle protein synthesis, and the number of nuclei is also positively correlated with myofiber size (Snijders et al., 2020; Ato and Ogasawara 2021), but muscle protein synthesis was negatively correlated with myofiber size (Ato and Ogasawara 2021). Scaling properties of adult human and mice muscle fibers are the same for myonuclei number and cell volume (Hansson et al., 2020). MND may differ between fiber types in mammals, where slow-oxidative fibers have a smaller MND compared with glycolytic fibers (Tseng et al., 1994; Liu et al., 2009; Van der Meer et al., 2011; Omairi et al., 2016). This difference originates from fewer myogenic nuclei in glycolytic fibers rather than a smaller fiber volume in slow-oxidative fibers (Van der Meer et al., 2011). Thus, MND is inversely related to oxidative capacity of the muscle fiber itself, because slow-oxidative fibers should need faster protein turnover rates than fast-glycolytic fibers (Tseng et al., 1994; Liu et al., 2009; Van der Meer et al., 2011; Omairi et al., 2016). Fast-glycolytic fibers demonstrate high flexibility in MND (Murach et al., 2018). Furthermore, manipulating Myostatin or Akt pathways that are associated with hypertrophy also increases fiber diameter without myonuclear accretion in fast-glycolytic fibers (Murach et al., 2018). SCs also respond to increases in IGF-1 by stimulating proliferation (Scicchitano et al., 2016; Forcina et al., 2019). Exercise in mammals typically promotes SC activation. In mice, after 4 weeks of training, SC activation can happen within 24 h of acute exercise (Wen et al., 2021).

### Myonuclear domain and muscle remodeling in birds with respect to temperature changes

Post-natal development of muscle fibers involves an increase in fiber size and a concomitant increase in the number of nuclei (derived from SCs) (Hughes and Schiaffino, 1999; Brack et al., 2005). In adult birds, SCs may proliferate following stretching (Winchester and Gonyea, 1992). In black-capped chickadees, when fiber diameter increases in colder seasons via hypertrophy, MND also increases (Jimenez et al., 2019). This indicates that each myonucleus must regulate synthesis and degradation for a greater area of the muscle fiber (Van der Meer et al., 2011). Additionally, this implies that increases in thermogenic capacity are coupled with remodeling of muscle tissue protein processing such that each myonucleus may need to respond to an increased demand for protein turnover. It is, however, possible that MND increases prior to SCs being incorporated into the myofiber. Cold-acclimated chickadees exposed to a sudden 15°C drop in temperature are able to modify their pectoralis ultrastructure within 3 h of the temperature decrease (Vézina et al., 2020). Within 3 h, these birds were able to increase the number of nuclei per millimeter of fiber by 15%, and decrease MND by the same amount. This may suggest that the addition of SCs into existing myofibers can be rapid (Vézina et al., 2020). Others have also demonstrated that cultured muscle SCs have rapid proliferation rates (Young et al., 2021a). After muscle injury, 73% of the myonuclei found at the periphery of the fiber migrated to the site of injury within 5 h in adult mice, thus, movement of nuclei within a myofiber seems to be rapid and dynamic (Roman et al., 2021). These additional nuclei may originate from either symmetrical or asymmetrical divisions of SCs (Kuang et al., 2007), as DNA content per myonucleus (or genome size) of avian muscle does not increase during muscle remodeling (Jimenez and Lencyk 2021). Thus, winter-phenotype chickadees facing a sudden cold drop may have activated SCs.

A further implication of these data is that, whereas avian muscle seems more phenotypically flexible than mammalian muscle, the biological processes surrounding myonuclear function may be more closely related to those of mammals (Jimenez 2020; Jimenez and Lencyk 2021). SCs have capacity for self-renewal and their ability to retain stem cell properties in mammals is well documented (Sobolewska et al., 2011; Shimizu-Motohashi and Asakura, 2014; Forcina et al., 2019; Abreu and Kowaltowski, 2020). Because ploidy number does not change in bird muscle tissue, phenotypic flexibility of avian muscle may be limited by self-renewal capacities of SCs (Jimenez and Lencyk 2021). If avian SCs are capable of self-renewal and act as stem cells, as they apparently do in mammals (Forcina et al., 2019), especially during exercise or exercise-induced injury, then activating new satellite cells into the muscle fiber under conditions promoting hypertrophy might not be a limiting factor for birds. Additionally, Pax7 (a marker for adult SCs) expression in aging chickens was not different from that in young chickens, highlighting that Pax7 protein expression does not decrease with age in birds as it does in mammals (Jankowski et al., 2020). This is consistent with proliferative capacities of SCs being maintained across the lifespan of birds (Brown et al., 2019).

Jimenez and De Jesus (2021a) found that fast-growing quail subjected to an acute temperature increase had lower numbers of nuclei per mm of fiber than control quail. In opposition, in house sparrow pectoralis muscle, higher numbers of nuclei per mm of fiber occurred in the control (winter phenotype) group compared with the heat-shocked and recovery group (De Jesus and Jimenez 2021). Both fast-growing quail and winter phenotype sparrows likely grow adult muscle fibers via hypertrophy. Thus, the control group of sparrows should have higher numbers of nuclei, compared with the heat-shocked and recovery group, as their winter phenotype pectoralis muscle should have undergone hypertrophic accretion, similar to fast-growing quail, suggesting a similar physiological mechanism of control. Additionally, tropical bird species (exposed to a thermally stable warm climate) had more nuclei per mm of muscle fiber compared with their temperate counterparts, but no differences in MND (Jimenez and De Jesus 2021b).

Changing environmental temperatures can affect chick muscle development (Modrey and Nichelmann, 1992), which is mediated by SCs. The neonatal period in chick muscle growth is when SCs are most active (Halevy et al., 2000). SC proliferation increases with temperature during late embryogenesis or early life in chicks (Halevy et al., 2000; Loyau et al., 2013; Piestun et al., 2017), yielding increases in muscle mass. SCs isolated from turkeys and exposed to differing environmental temperatures also demonstrated increased proliferation rates with increased temperatures, but decreased proliferation and differentiation rates at ambient temperatures lower than control values of 38°C (Clark et al., 2016). Furthermore, just 24 h of transitory heat stress in a chick’s first week of life promoted increased SC proliferation and differentiation (Halevy et al., 2000). Mild heat stress, even at 3 weeks of age, may also increase SC proliferation into existing myotubes, at least in birds with slower growth rates (Jimenez and De Jesus 2021a). The reason SC profiferation may not be the same in faster-growing birds could result from either the availability of SCs, the environment in which SCs occur (Murach et al., 2018; Forcina et al., 2019), or dramatic increases in muscle fiber size without proportional addition of myonuclei (Velleman et al., 2003).

SC activities including proliferation and differentiation are highly responsive to environmental temperatures, especially early in life (Halevy et al., 2006; Xu et al., 2022), suggesting that muscle fiber diameter and temperature are related (Clark et al., 2016; Xu et al., 2021). Cultured adult turkey pectoralis major muscle SCs demonstrated heightened proliferation and differentiation when temperature was increased from 33 to 43°C. Thus, temperature increases seem to be a signal for avian SCs to increase activity, proliferation and *MyoD* expression (Clark et al., 2016; Xu et al., 2021). However, this may happen over a series of days and it is most pronounced within the first week of life. In contrast, 24 h of heat shock in adult house sparrows reduced the number of nuclei per millimeter of fiber and increased MND, both of which were not corrected by 24 h of recovery (De Jesus and Jimenez 2021). This implies that the timing of thermal challenges may be important with respect to the response of SCs within the avian pectoralis muscle. Secondly, the SCs could have been partially activated by the heat, but instead lacked the proper environment and vasculature needed for full activation, termed the muscle stem cell niche (Velleman, 2015). Additionally, thermal stress can change SC proliferation and differentiation through the mTOR/S6K pathway with faster-growing birds showing greater SC activity compared to those with slower growth rates (Xu et al., 2022).

### Myonuclear domain and muscle remodeling in birds with respect to flight exercise

Besides thermally induced remodeling in avian muscle, the number of nuclei per fiber is positively associated with flight velocity in black-legged kittiwakes, likely because higher power output needed by faster-flying birds required plasticity for muscle fiber recruitment (Lalla et al., 2020). The number of nuclei per muscle fiber increases with muscle training, even preceding hypertrophic muscle growth (Bruusgaard et al., 2010), supporting our hypothesis that a higher number of nuclei per fiber is associated with faster flight speeds. It should be noted, however, that the hypothesis that muscle has “memory” after training such that nuclei are maintained after detraining has been challenged (Dungan et al., 2019; Murach et al., 2020). Research in this area of study is severely lacking and would be extremely informative.

### Potential mechanisms driving MND flexibility in birds

Flexibility around MND, including hypertrophic growth (Murach et al., 2018), appears to be the mechanism employed by avian muscle in response to decreases in temperature and to migration (Jimenez 2020). Such hypertrophic and MND responses appear to be dominated by the number of nuclei within a fiber. Acquiring new nuclei may benefit muscle function more than upregulating the output of existing nuclei when MND is increased (Cramer et al., 2020). Thus, mechanisms of avian muscle flexibility, including increases in muscle mass and muscle fiber diameter, may involve mammalian-like processes (Jimenez 2020). For example, it is generally assumed that SC-dependent myonuclear accretion, which is required for adult skeletal muscle hypertrophy, is the first step for increasing muscle fiber diameter, though hypertrophy may occur without SC addition (Murach et al., 2018). SC fusion within a myotube increases DNA content, but how this relates to muscle fiber protein output is unclear (Kirby et al., 2016). When DNA content is held constant, however, myonuclei upregulate transcriptional activity (Kirby et al., 2016). It is generally assumed that fiber diameter regulation is related to protein turnover potential in muscle fibers, such that fiber diameter is positively correlated with protein turnover rates (Kirby et al., 2016; Figueiredo 2019). Muscle hypertrophy in response to exercise results from repeated short-term increases in protein synthesis (likely of myofibril proteins) following each bout of exercise (Figueiredo 2019). These increases in RNA content during hypertrophy are likely due to increases in transcriptional activity (Kirby et al., 2016). That birds increase muscle fiber diameter not only for migratory flights or in response to decreases in temperature, but prior to winter or migration may suggest that muscle growth may be dictated by differing mechanisms in birds than in mammals (Driedzic et al., 1993). Hypertrophic adaptation by mechanical stimuli (exercise) may fundamentally differ from non-mechanically mediated hypertrophy (growth) (Murach et al., 2018). It would be of great interest to develop a framework to tease apart mechanistic differences between these two modes of hypertrophic muscle growth in birds.

Regarding the relationship between SCs and muscle remodeling pathways, studies addressing the relationship between myostatin and SCs proliferation in mammals provide mixed results. For example, some studies suggest that muscle hypertrophy driven by decreases in myostatin does not result in more nuclei per fiber and SC proliferation rates do not change in the absence of myostatin (Amthor et al., 2009). Others have found that myostatin inhibits SC proliferation and self-renewal (McCroskery et al., 2003). In contrast, increases in IGF-1 seem to increase the activation of SCs (Machida and Booth, 2004). How these two pathways affect SC activation and proliferation in adult wild birds warrants future study (Duclos et al., 1999; Halevy et al., 2001).

## Conclusion and future directions

In summary, avian muscle remodeling is a common component of flexible responses to changes in energy demand, such as cold temperatures or migratory flights. Studies focusing on the signaling pathways regulating avian muscle remodeling, however, are still limited, inconclusive and biased towards myostatin. Strategies and the underlying signaling pathways employed by birds might be species- or context-specific (Zhang et al., 2018a). In order to cope with such complexity, we first encourage researchers to employ an integrative approach to study these signaling pathways and phenotypes they produce at a variety of biological levels (Zhang and Wong, 2021). Moreover, using genetic manipulation of primary avian muscle cells (Young et al., 2021a; Young et al., 2021b), or pharmaceutical activators/inhibitors (such as follistatin for myostatin) might provide alternative and/or more comprehensive answers. Secondly, intracellular signaling pathways controlling muscle mass, such as the Akt/TSC2/mTOR pathway (Saxton and Sabatini, 2017), have also received limited research attention relating to phenotypic flexibility in wild birds. Further study targeting genes in these pathways under conditions of increased energy demand in wild birds seems warranted. Moreover, studies to date on myostatin or IGF-1 and phenotypic flexibility in wild birds are correlative in nature, so functional roles for myostatin and IGF-1 in flexible responses of muscle to changes in energy demand have yet to be demonstrated. Future studies experimentally manipulating myostatin or IGF-1 levels in wild birds followed by examination of changes in SCs, MND, mitochondria, muscle fiber diameter, muscle mass and organismal metabolic capacities would be useful in validating roles for myostatin and IGF-1 pathways in regulating muscular and metabolic flexibility in wild birds. Thirdly, besides regulating protein synthesis, skeletal muscle mass can also be regulated through transduction pathways that control protein degradation. Situations altering the synthesis/degradation balance of myofibrillar proteins may thus contribute to muscle hypertrophy or atrophy. Unfortunately, pathways associated with protein degradation in muscles, such as ubiquitin proteasome system and FOXO-mediated signals have not been investigated in the context of avian phenotypic flexibility.

**FIGURE 3:**
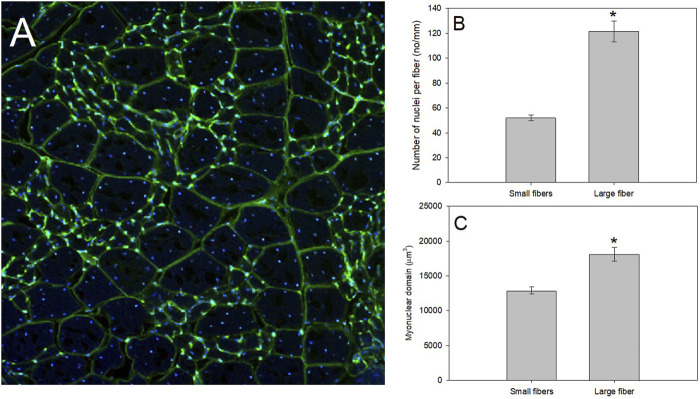
A productive model system to study regulation of muscle fiber size as whole-animal energetics change for birds is that of the pectoralis of mourning doves. **(A)** After fixing the pectoralis muscle in 4% paraformaldehyde, we placed fixed muscle tissue in 25% sucrose for 24 h to cryo-protect the samples. Tissues were then flash frozen in isopentane cooled in liquid nitrogen, mounted at resting length in Optimal Cutting Temperature (O.C.T.) compound and allowed to equilibrate to −19°C in a Leica 1800 cryocut microtome before sectioning. Sections were cut at 30 μm, picked up on slides, air-dried at room temperature, stained with a 250 μg/ml solution of wheat germ agglutinin (WGA) labeled with Alexa Fluor 488 (in green), and 4′,6-diamidino-2-phenylindole (DAPI; in blue), for 30 min, and rinsed in avian ringer’s for 60 min. WGA is a lectin that binds to glycoproteins on the basement membrane of the fiber sarcolemma, and effectively outlines the fiber periphery to allow measurements of fiber size, whereas DAPI irreversibly binds to nuclei. Stained slides were examined with an Olympus Fluoview 1000 laser filter confocal microscope, and pictures were taken at a magnification of ×20. Mourning dove pectoralis muscle contain a population of small muscle fibers with a myonuclear domain (MND) surrounded by a population of large muscle fibers. **(B,C)** Using data from Jimenez and De Jesus (2021b), we isolated the number of nuclei per fiber and MND of *N* = 4 mourning doves (*N* = 135 small fibers and *N* = 63 large fibers). Using a one-way ANOVA, the small fibers demonstrated a significantly fewer nuclei per mm of fiber (*F* = 108.83, *p* < 0.0001; Panel **(B)**, and a significantly smaller MND (*F* = 27.48, *p* < 0.001; Panel **(C)**.

MND-related questions in the avian study model are still in a primitive state, with only a few studies addressing this concept. Regulation of MND in birds appears to be dominated by some of the same mechanisms as mammals, however, its ability to adjust to thermal or workload changes may be faster, potentially due to avian SCs ability to proliferate quickly (Young et al., 2021a). Future studies in this field would benefit from addressing communication of these exogenous cues across the myotube. Myonuclei in the syncytium may organize by dividing transcriptional labor to achieve specific functions (Wen et al., 2021), but how these functions are shared across the myotube is unclear. Thus, experiments surrounding the activation of myonuclei with differing stressors (cell-wide vs. sarcolemmal, for example), would be valuable in deciphering protein expression patterns within the myotube. An interesting avian study model to address this question is mourning dove (*Zenaida macroura*), which demonstrated a pectoralis muscle fiber population including very small and very large muscle fibers (Figure 3). These size differences within one muscle tissue should allow studies of the functionality of a syncytium and how MND labor is dictated. Additionally, determining how regulation to either add nuclei or upregulate existing nuclear activity and under what ecological or environmental conditions such changes occur in birds would be another fruitful topic for study (Machida and Booth, 2004; Noakes and McKechnie, 2020; Attwaters and Hughes, 2021; Snijders et al., 2021).
